# A Review on Xerostomia and Its Various Management Strategies: The Role of Advanced Polymeric Materials in the Treatment Approaches

**DOI:** 10.3390/polym14050850

**Published:** 2022-02-22

**Authors:** Afroditi Kapourani, Konstantinos N. Kontogiannopoulos, Alexandra-Eleftheria Manioudaki, Athanasios K. Poulopoulos, Lazaros Tsalikis, Andreana N. Assimopoulou, Panagiotis Barmpalexis

**Affiliations:** 1 Department of Pharmaceutical Technology, School of Pharmacy, Aristotle University of Thessaloniki, 54124 Thessaloniki, Greece; akapourag@pharm.auth.gr (A.K.); kkontogi@cheng.auth.gr (K.N.K.); alexandme@pharm.auth.gr (A.-E.M.); 2 Department of Oral Medicine and Maxillofacial Pathology, School of Dentistry, Aristotle University, 54124 Thessaloniki, Greece; akpoul@dent.auth.gr; 3 Department of Preventive Dentistry, Periodontology and Implant Biology, School of Dentistry, Aristotle University of Thessaloniki, 54124 Thessaloniki, Greece; tsalikis@dent.auth.gr; 4 Laboratory of Organic Chemistry, School of Chemical Engineering, Aristotle University of Thessaloniki, 54124 Thessaloniki, Greece; adreana@cheng.auth.gr; 5 Natural Products Research Centre of Excellence-AUTH (NatPro-AUTH), Center for Interdisciplinary Research and Innovation (CIRI-AUTH), 57001 Thessaloniki, Greece

**Keywords:** xerostomia, artificial saliva, salivary substitutes, salivary stimulants, advanced polymers, mucoadhesive

## Abstract

The medical term xerostomia refers to the subjective sensation of oral dryness. The etiology seems to be multifactorial with the most frequently reported causes being the use of xerostomic medications, neck and head radiation, and systematic diseases (such as Sjögren’s syndrome). Xerostomia is associated with an increased incidence of dental caries, oral fungal infections, and difficulties in speaking and chewing/swallowing, which ultimately affect the oral health-related quality of life. The development of successful management schemes is regarded as a highly challenging project due to the complexity of saliva. This is why, in spite of the fact that there are therapeutic options aiming to improve salivary function, most management approaches are alleviation-oriented. In any case, polymers are an integral part of the various formulations used in every current treatment approach, especially in the saliva substitutes, due to their function as thickening and lubricating agents or, in the case of mucoadhesive polymers, their ability to prolong the treatment effect. In this context, the present review aims to scrutinize the literature and presents an overview of the role of various polymers (or copolymers) on either already commercially available formulations or novel drug delivery systems currently under research and development.

## 1. Introduction

Xerostomia (or dry mouth) is the medical term used to describe the subjective sensation of oral dryness, which commonly exists as a consequence of reduced salivary flow (hyposalivation) [1,2,3]. However, despite its connection to salivation, studies have shown that in various cases, patients with xerostomia appear to have normal salivary flow [4,5,6]. Hence, the term “symptomatic” xerostomia (or else “pseudo” xerostomia) is nowadays used to refer to oral dryness despite the salivary gland function [7,8,9]. In general, patients with xerostomia suffer symptoms that significantly affect their health as well as social and emotional aspects of their life. Currently, the diagnosis and therapeutic approaches of this condition vary, while it is difficult to achieve favorable results, since the etiology seems to be multifactorial. The majority of the management options aim to relieve oral discomfort by keeping mouth moisture at an acceptable level. In most of these therapeutic scenarios, polymers are an integral part of the different formulations used at every current treatment approach (as discussed in detail hereunder) due to their pivotal role as thickening and lubricating agents, while mucoadhesive polymers (i.e., polymers, synthetic or natural, which are capable of attaching to mucosal surfaces) are frequently used for prolonging the treatment effect. In this vein, this review sets the foundations for the identification of the polymer’s exact task on the xerostomia’s treatment. Characteristically, specific examples of commercially available products with a polymeric base and innovative drug delivery systems currently under research are provided in order to establish the vital role of polymers at the development of various management schemes, whether in commerce or in the research field. Furthermore, the specific properties that polymers attribute to the products are revealed.

## 2. Approach of the Review

The aim of this review was to scrutinize the literature regarding xerostomia and salivary gland hypofunction and, specifically, the underlying causes, the impact on quality of life, and the recommended management approaches. Significant attention was paid especially to the polymers’ role in the various therapeutic approaches. A literature search was conducted during September 2021 to January 2022, utilizing the electronic databases of MEDLINE/PubMed, SCOPUS, and Google scholar, for original research articles in the English language, already published, or at “in press” status in peer-reviewed literature. The terms “xerostomia”, “saliva substitute”, “artificial saliva”, “salivary stimulation”, “cholinergic agents”, “pilocarpine”, “cevimeline”, “xerostomia drug formulations”, and “dry mouth drug formulations” were used. Related links until 2021, as well as articles referenced in the initially retrieved papers, have also been taken into consideration and were included if pertinent. After a careful analysis of the output of these searches, 132 articles fitted our criteria, as referenced below, and were included in this review.

In regard to the management of xerostomia, various management approaches of xerostomia, concerning either already commercially available formulations or delivery systems currently under research and development, have been taken into consideration. Additionally, the crucial role that polymers play in the development of the various management approaches in this research is indisputable, being referred to in 68% of the 63 studies comprised in the section for disease management.

## 3. Saliva Production in Humans

**Figure 1:**
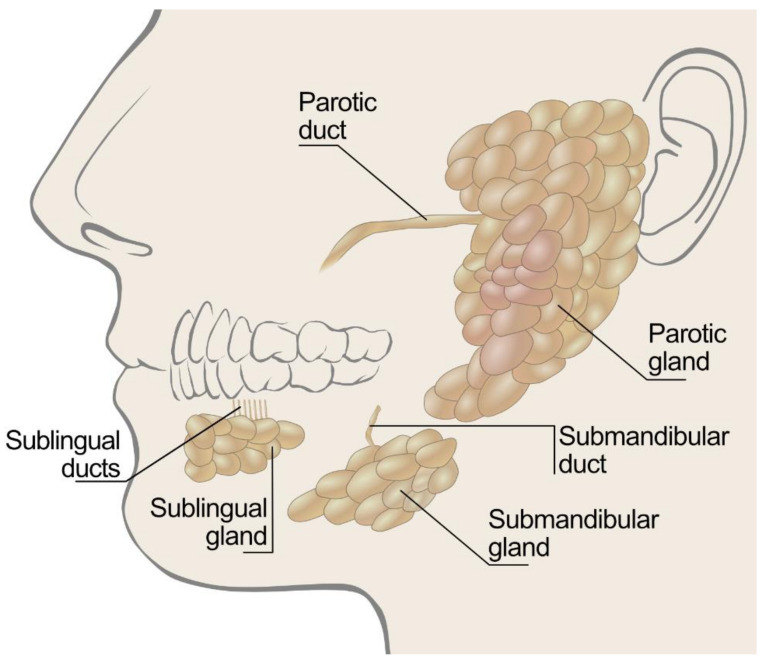
Illustration of salivary glands types and position.

Saliva is a very complex fluid that is mainly composed of water combined with electrolytes, minerals, buffers, growth factors, enzymes, cytokines, proteins, and immunoglobulins [10,11,12,13]. In humans, it is produced from the major and minor salivary glands of the mouth, as depicted in Figure 1.

Ninety percent of the average daily salivary secretion (≈1–1.5 L) is produced by the major salivary glands (this is the parotid, submandibular, and sublingual glands), while the minor salivary glands spontaneously produce the remaining 10% of the total salivary secretions [12,14]. The minor glands are important, as their ducts open onto most areas of the oral mucosa except for the area covering the dorsum of the tongue, the anterior part of the hard palate, and the gingiva. They can be grouped into lingual, labial, buccal, palatine, and glossopalatine [1]. The secretions from the salivary glands are innervated by both the parasympathetic and sympathetic nervous systems [15,16]. Specifically, when parasympathetic innervations dominate, the secretions are more watery, whereas the sympathetic system produces a more viscous flow, since the secretions contain more proteins from acinar cells [17]. Therefore, a sensation of dryness may occur, for example, during episodes of acute anxiety or stress, which cause changes in salivary composition owing to predominant sympathetic stimulation during such periods [18]. In general, there is great variability in the salivary flow rate for each individual. The average unstimulated whole salivary flow rate is 0.3 to 0.4 mL/min during waking hours, with an unstimulated flow rate below 0.1 mL/min indicating hyposalivation [19,20,21,22].

## 4. Diagnosis of Xerostomia

Xerostomia is often referred as hyposalivation [4]; however, these two terms do not correspond to identical conditions and should not be used interchangeably. Hyposalivation refers solely to the objective observation of reduced salivary flow due to external or internal influences, while xerostomia encompasses the subjective sensation of oral dryness [23]. The clinical method most often employed for the diagnosis of salivary dysfunction is the sialometry test, in which hyposalivation is considered to appear when salivary flow rates are under 0.1 mL/min at rest (UWS) or 0.7 mL/min under stimulation (SWS) [24].

A systematic approach is used to distinguish patients with symptoms of xerostomia, using measurable salivary gland hypofunction. The diagnosis of xerostomia requires a thorough medical history, which includes a detailed description of the symptoms (patients with xerostomia often complain of a dry and sticky sensation in the mouth, which makes it considerable difficulty to swallow and speak, while a decrease in taste sensation might also be presented) and of the medication used [25,26,27]. Several scientifically validated questionnaires have been designed specifically to evaluate a possible salivary glandular dysfunction and xerostomia [5], among which the questionnaire developed by Fox et al. [28,29,30] is the most frequently used to take the medical history of patients. Salivary hypofunction could also be diagnosed using four additional clinical measures: dryness of the lips, dryness of the buccal mucosa, absence of saliva produced by gland palpation, and total decayed–missing–filled teeth (DMFT) [31].

## 5. Causes of Xerostomia

Depending on their nature, the causes of xerostomia can be classified as systemic or local [32]. Based on the duration of the symptoms, the condition is qualified as persistent or periodic. Systemic causes of xerostomia include endocrinological (e.g., diabetes mellitus, autoimmune thyroid diseases), autoimmune (e.g., Sjögren’s syndrome, rheumatoid arthritis, systemic lupus erythematosus), infectious (e.g., hepatitis C virus), and granulomatous (e.g., tuberculosis and sarcoidosis) diseases. Local factors that are recognized as responsible for xerostomia are multiple medications (polypharmacy), radical radiotherapy for treatment of head and neck malignancies, and lifestyle factors, such as alcohol, tobacco, and caffeine consumption [33,34,35]. Interestingly, a correlation between xerostomia and the recent coronavirus disease-19 (COVID-19) has been reported. Specifically, according to the results of the study conducted by Fantozzi et al., 45.9% of patients with confirmed SARS-CoV-2 infection suffered xerostomia, with a significant majority (76.5%) of them mentioning that it was their first-time experiencing xerostomia in their lifetime [36].

### 5.1. Local Factors

Polypharmacy (the prescription of multiple medicines) is regarded as the most common cause of xerostomia, which in some way explains the aforementioned association between the elderly population and the prevalence of dry mouth, since chronic diseases and multi-morbidities of geriatric patients result in widespread polymedication [37,38]. From 2005 to 2011, in the United States, more than one-third of older adults used ≥5 prescription medications concurrently [39], and more than 75% of people over the age of 65 took at least one medication prescription that may affect salivary function [40]. It is estimated that more than 400 medications favor the occurrence of xerostomia and affect the salivary gland function [41]. Although the exact mechanisms by which some drugs cause xerostomia are still unknown, the common offenders of xerostomia include antiparkinsonian medications, antipsychotic agents, antidepressant medications, diuretic agents; opioids; cytotoxic agents, and antihypertensive medications [42,43,44]. While drug-induced xerostomia is generally reversible, the conditions for which these medications are prescribed are frequently chronic. A detailed list of agents that cause drug-related xerostomia is presented in Table 1.

Radiotherapy is one of the prominent integral components in the multidisciplinary management of head and neck cancer, yet it produces considerable acute and long-term side effects. One of the most frequent complications of conventional radiotherapy is xerostomia, since the major salivary glands are usually included in the radiation portals [46,47,48,49]. Specifically, it is assumed that the radiation exposure harms the blood vessels or nerves supplying these glands and not the salivary glands themselves [50]. However, there is a consensus that xerostomia is sufficiently limited by keeping the mean dose to the total parotid volume below 26 Gy [51].

### 5.2. Systematic Diseases

Sjögren’s syndrome is an autoimmune disease characterized by the inflammation of the exocrine glands, mainly of the lacrimal and salivary glands [52,53]. The two categories of the disease are the primary form, which is characterized by the independent occurrence of the disease, and the secondary form, which is associated with other autoimmune diseases, such as rheumatoid arthritis, scleroderma, and systemic lupus erythematosus. Some common oral manifestations of Sjögren’s syndrome are mainly xerostomia and hyposalivation, autoimmune sialadenitis, and dental caries [54]. The xerostomia that is associated with primary and secondary Sjögren’s syndrome has been attributed to the progressive lymphocytic infiltration that gradually destroys the secretory acini of the major and minor salivary glands.

Diabetes mellitus is a chronic multi-systemic metabolic disease characterized by hyperglycemia due to either a deficiency of insulin secretion or resistance to the action of insulin or both [55,56]. The oral manifestations and complications related to diabetes mellitus include, among others, xerostomia, tooth decay, gingivitis, periodontal disease, oral candidiasis, burning mouth, and altered taste [57,58]. Researchers have identified a bidirectional adverse relationship between diabetes and oral diseases [59]. Diabetic patients suffer from xerostomia and salivary gland hypofunction, which may be related to polydipsia and polyuria, autonomic neuropathies, and alterations in the basement membranes of salivary glands.

**Figure 2:**
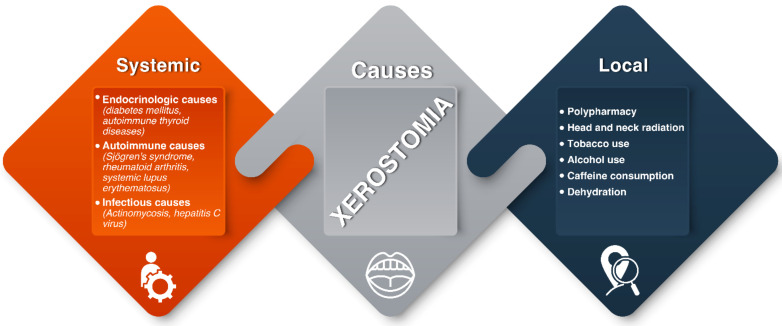
The classification of xerostomia’s causes as systemic or local.

All the aforementioned xerostomia’s reasons are summarized in Figure 2.

## 6. Effects of Xerostomia

Although xerostomia can affect a person at any age, it appears to be most prevalent in postmenopausal women and the elderly population [60]. In a study of over 5000 individuals, Johansson et al. [61] examined the prevalence, the progression, the yearly incidence of xerostomia, and its effect on 50- to 80-year-old people. In all age groups, xerostomia was significantly more prevalent in women than in men; the prevalence increased with age and was more frequent during night-time. Xerostomia is associated with an increased incidence of dental caries, oral fungal infections (e.g., candidiasis), halitosis or burning mouth, and periodontal disease. Furthermore, clinical effects include dysphagia, dysgeusia, and difficulty in speaking, chewing, and swallowing, which ultimately affect oral health-related quality of life (OHRQOL) [50,62,63,64,65,66]. Additionally, looking at the literature, a correlation between decreased salivary flow rate and low nutritional assessment score is suggested, as determined by body mass index (BMI), mid-arm circumference, triceps skinfold thickness, and serum albumin level [67,68].

## 7. Management of Xerostomia

The establishment of the correct diagnosis is considered as the most crucial step in the management of patients with xerostomia, since it encompasses the distinguishment of patients with subjective complaints from those presenting salivary gland hypofunction as well [4]. Once a diagnosis is established and an underlying etiology is identified, a stepwise management approach can be implemented, aiming to institute preventive measures, alleviate symptoms, treat oral manifestations, and improve salivary function.

### 7.1. Preventive Approaches

In a first step, preventive measures must be followed by every patient who suffers from xerostomia in order to prevent the development of oral infections associated to the disorder. Specifically, diligent oral hygiene and regular dental care—with examinations very 4–6 months—are essential [69]. It is also important to inform patients about the role of dietary sugars and refined carbohydrates in the development of caries, so their intake is minimized or discouraged [1,70,71]. Furthermore, a topical application of fluorides (e.g., fluoridated toothpaste, daily fluoridated mouth washes, and application of fluoridated gel) is also beneficial for the management of hyposalivation-induced caries, especially in cases of patients whose xerostomia has resulted from radiation therapy to the head and neck [72,73,74,75].

### 7.2. Symptomatic Relief: Salivary Substitutes

The symptomatic relief or control of oral dryness includes hydration (frequent sipping of water), discontinuation or reduction in xerogenic medications, and elimination of common dry mouth offenders, such as tobacco and alcohol [76]. Moreover, artificial salivary substitutes (i.e., commercial products containing specific ingredients, whose properties resemble those of the natural saliva) are frequently used as symptomatic treatments for patients with decreased salivary flow rate [77,78]. In fact, they act as oral lubricants that maintain the lubrication of the mucosa and, hence, relieve the sensation of dryness, without stimulating the salivary flow. However, it should be pointed out that saliva substitutes’ action present limited duration and, therefore, a frequent re-application is required, which creates issues around patient adherence and increases the cost of therapy.

An ideal salivary substitute should resemble the properties of human saliva and, simultaneously, provide a pleasant taste aiming to a convenient self-administration and increased patient compliance [79,80]. The development of artificial saliva requires in-depth understanding of both biological and rheological (e.g., viscosity and film-forming wettability) properties of human saliva, which is composed of a mixture of macromolecules. Specifically, human saliva is regarded as a non-Newtonian fluid, because of the salivary glycoproteins’ presence. This characterization means, in essence, that saliva’s viscosity varies, depending on the shear rate [81]. Thus, the efficacy of artificial saliva as a lubricant is partially dependent on its viscosity and how this changes with shear rates [82]. Since different shear rates may be present in the oral cavity—from 60 to 160 s^−1^ during processes such as swallowing and speaking—the high importance of the aforementioned phenomenon is apparently highlighted [83]. So, salivary substitutes are expected to have a viscoelastic pattern similar to normal human saliva in order to provide similar viscosity and film-forming properties. Taking into consideration that the principal aim of saliva substitutes is to ensure the lubrication of oral tissues, it is obvious that apart from the viscosity, lubrication is considered as another important factor for the clinical acceptance of saliva substitutes. Concerning the biological properties of artificial saliva, substances of natural origin, including enzymes such as lysozyme and peroxidase or proteins such as lactoferrin and mucin, may be utilized in order to provide high biocompatibility [84].

**Figure 3:**
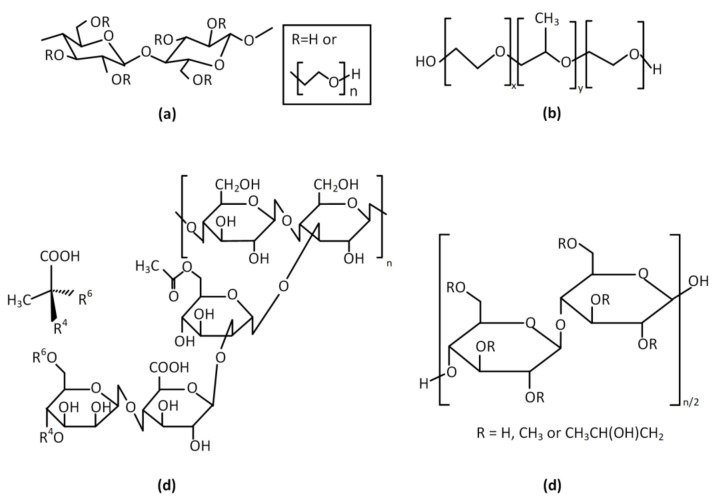
Structures of commonly used polymers for artificial saliva: (**a**) hydroxy ethylcellulose (HEC); (**b**) poloxamer or Pluronic^®^ (PEO-PPG-PEO); (**c**) xanthan gum; (**d**) hydroxypropyl methylcellulose (HPMC).

Different dosage forms are available in the market such as cleansers, gels, sprays, and lozenges. The majority of the commercial salivary substitutes are commonly based on animal mucin or on polymeric thickening and moisturizing agents, such as cellulose-based polymers (e.g., carboxymethylcellulose (CMC), hydroxyethylcellulose (HEC), hydroxypropylmethylcellulose (HPMC) [85,86]) and water-soluble polymers, such as xanthan gum and carbomer [87]. The referred polymers are integrated in saliva substitutes, since they provide certain properties at formulations and, hence, fulfill the significant aforementioned standards. Specifically, CMC—the most commonly used polymer in the saliva substitutes’ development, even though it is not a natural lubricant—has been proved as a decent clinical choice for the basis of a saliva substitute, improving a formulation’s viscoelastic properties. It was shown that the wetting properties of CMC-containing saliva substitutes on human enamel were significantly better than those of human whole saliva and comparable with those on human oral mucosa [88]. Moreover, a prospective cross-over study in patients with xerostomia comparing four different polymers used in saliva substitutes showed that the majority of patients preferred the CMC-based product due to its palatability and easy handling. Moreover, saliva substitutes containing other cellulose derivatives (i.e., sodium carboxymethyl cellulose (SCMC), methyl cellulose (MC), and HPMC) have been prepared. The examined polymers provide to final formulations some physical properties resembling those of human saliva, rendering them high-quality standard formulations [89]. As for xanthan, it is an anionic biopolymer with repeated chains of cellulose monosaccharides and oligosaccharides, which is utilized in the pharmaceutical industry due to its tunable thickening, stabilizing, suspending, and emulsifying properties. Characteristically, the most noticeable xanthan’s property is its very high low-shear viscosity coupled with its strongly shear-thinning character. Relatively low viscosity at high shear means that it is easy to mix, pour, and swallow, but its high viscosity at low shear gives decent suspension and coating properties and lends stability to colloidal suspensions. These features could explain why xanthan gum-containing saliva substitutes have presented synergistic effects on the elastic and rheologic properties of human whole saliva [90]. The structures of the most commonly used polymers at saliva substitutes are presented in Figure 3.

Each formulation differs from another in respect to the base substance, the chemical composition, and the viscosity. Studies have shown that the viscosity of mucin-based saliva substitutes resembles natural saliva more closely than formulations based on CMC or polyethylene oxide; notwithstanding, there is no evidence regarding whether one formulation is superior to another [91]. Patients should select different products based on the severity of xerostomia, their daily routine and, even, the time of the day [92]. Characteristically, in severe xerostomia, a gel-like salivary substitute should be used overnight, whereas a more liquid substitute is recommended as more appropriate during the day.

In any case, polymers play a crucial role in the saliva substitutes’ development and, specifically, in the exact property of mimicking the techno-functionalities of real human saliva. This is confirmed by the fact that a polymeric base substitute is trusted to be used in numerous commercially available salivary substitutes. Table 2 summarizes different commercial saliva substitutes and highlights the polymers that are utilized by these formulations.

### 7.3. Salivary Stimulation

One point to be highlighted here is that salivary substitutes are utilized when salivary glands are completely damaged. In case there is residual functional salivary tissue, one of the alternatives for xerostomia and hyposalivation is the use of salivary stimulants [100,105]. Generally, salivary stimulation can be divided into acid-, pharmaceutically-, and mechanically- driven [77].

Acid-driven stimulation of salivary secretion is generated by the acidification of the oral cavity, with malic and citric acid being the most commonly preferred sialogogues acid [106]. Mechanical salivary stimulation, on the other hand, includes the utilization of chewing gums—usually sugar-free and artificially sweetened with aspartame and sorbitol—acupuncture, and electrostimulation [107,108,109,110]. In xerostomia, sugarless chewing gums are used to stimulate the major salivary glands, aiming to increase the saliva secretion through mechanical stimulation and decrease the oral mucosal friction [107,111,112]. The stimulation of the saliva secretion also increases the plaque pH, reducing the risk of caries formation [113]. A gum composition includes a water-soluble bulk portion and a water-insoluble gum base, consisting of several ingredients such as fillers, elastomers, emulsifiers, sweeteners, flavoring, and texture-regulating agents. The gum base can be natural or synthetic, composed mostly of elastomers, which are polymers providing elasticity and flexibility to the gum formulation [113]. Table 3 presents the polymers used in chewing gums’ formulations, while Table 4 refers to some commercially available chewing gums for xerostomia and their presented characteristics.

As far as the pharmaceutical approach is concerned, the administration of cholinergic agents, such as the parasympathomimetics and muscarinic agonists pilocarpine and cevimeline, in order to stimulate residual glandular function is widely implemented [123,124,125]. However, pharmaceutical stimulation may result in systemic adverse effects and, consequently, limited patient acceptance. Specifically, the use of orally administrated pilocarpine is contraindicated in patients suffering from gastric ulcer and uncontrolled asthma, while the risk of cardiovascular effects associated to the systemic administration is also a matter worth taking into consideration [126,127]. Cevimeline is a salivary gland stimulant with a stronger affinity for M3 muscarinic receptors. Since it has no effect on M2 receptors, it is expected to present fewer adverse effects when compared to pilocarpine [69,128]. However, clinical trials revealed similar adverse side effects to between cevimeline and pilocarpine.

**Figure 4:**
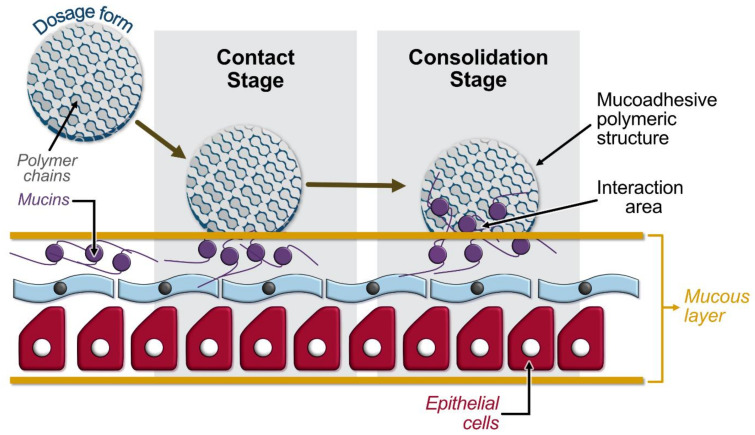
Illustration of the two-stage (contact and consolidation stage) mucoadhesion model.

Although the systemic administration of the cholinergic agents has been characterized by success, because of the increased risk of side effects, there is an urgent need to design novel and effective dosage forms presenting adhesive and sustained release properties for on-site demand to the intraoral surface. As a result, recent studies focusing on xerostomia’s treatment have turned their interest to mucoadhesive polymers and mucoadhesive dosage forms. Briefly, mucoadhesion is commonly defined as the adhesion between two materials, one of which, at least, is a mucus membrane. It can be affected by a number of factors, including hydrophilicity, molecular weight, cross-linking, swelling, pH, and the concentration of the active polymer. Mucoadhesive polymers have numerous hydrophilic groups, such as hydroxyl, carboxyl, amide, and sulfate. These groups attach to mucus or the cell membrane by various interactions such as hydrogen bonding and hydrophobic or electrostatic interactions [129]. The mechanism by which mucoadhesion takes place comprises two stages: the contact (wetting) stage, which is characterized by the initiation of interaction between the mucoadhesive polymeric and the mucous membrane, followed by the consolidation stage that involves interpenetration or entanglement of the polymeric and mucin chains [130]. Figure 4 presents an illustration of the aforementioned mechanism. Oral mucoadhesive drug delivery systems have received a great deal of attention for their potential to optimize localized drug delivery, since the oral mucosa is easily accessible and highly vascularized by a relative fast blood flow, allowing a direct access to the systemic circulation, bypassing the liver first-pass effect with consequent high bioavailability and acceptability by the patient [131]. Moreover, the oral mucosa is less susceptible to damage or irritation potentially related to drugs or excipients used, since it is characterized by a rapid cellular turnover (5–6 days).

**Figure 5:**
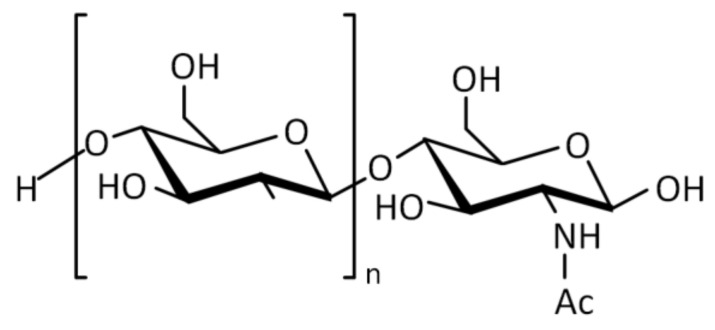
Structure of chitosan.

Looking at the literature, recently published studies provide a clear indication of the promising properties of chitosan as a mucoadhesive polymeric material for the preparation of novel xerostomia-treating formulations. Chitosan (Figure 5) is a cationic polysaccharide derived from chitin by partially deacetylating its acetamido groups with strong alkaline solutions [132]. Over the last two decades, it has been used for various biomedical and drug delivery applications due to its low toxicity, good biocompatibility, and mucoadhesive properties [133]. Chitosan has been reported to show excellent mucoadhesion on buccal mucosa, which makes it a promising candidate for the development of formulations aiming at the treatment of xerostomia.

In the case of xerostomia, Laffluer et al. [134] investigated the synthesis of novel preactivated chitosan conjugates and the development of a buccal adhesive semisolid dosage form comprising pilocarpine for patients with xerostomia. Specifically, unmodified chitosan was covalently linked to sulfhydryl possessing mercaptonicotinic acid (MNA) via the formation of amide bond. As for the safety profile, according to the obtained results from the carried out cell viability assay, no cytotoxicity was presented. Furthermore, mucoadhesion was improved in the presence of preactivated chitosan, and pilocarpine showed a controlled drug release in the presence of chitosan–MNA–MNA. The aforementioned observations, which might be attributed to the polymeric stability, render preactivated chitosan–MNA–MNA as a promising solution for the treatment of xerostomia.

Liposomes are nano-sized spherical vesicles composed of a lipid bilayer membrane that are able to encapsulate water-soluble molecules in their aqueous core. They have gained increased interest as carriers of active pharmaceutical ingredients, since they can present high biocompatibility and provide sustained drug release [135]. Furthermore, liposomes have been investigated in dental tissue regeneration, providing promising results [136]. The long-term stability of liposomes can be immensely improved by coating them with various polymers. In this context, Adamczak et al. [137] prepared different types of liposomes coated with five different types of polymers (i.e., low-methoxylated pectin (LM-pectin), high-methoxylated pectin (HM-pectin), alginate, chitosan, and hydrophobically modified ethyl hydroxyethyl cellulose (HM-EHEC)) and studied their efficacy on relieving dry mouth symptoms. Coating the liposomes with polymers significantly improved the water sorption capacity of the formulations in all cases. It is worth mentioning that the highest water sorption capacity along with a high mucoadhesion to the mucus-producing cells appeared in the case of the chitosan coated liposomes, demonstrating that these formulations could be another possible selection for relieving dry mouth symptoms. In a similar context, the research team of Tsibouklis et al. [40] published a review highlighting the need for novel hydrogel formulations with an affinity for buccal cells aiming at the management of xerostomia. Once again, various mucoadhesive polymers, such as chitosan, play the major role at the design of these hydrogels.

**Figure 6:**
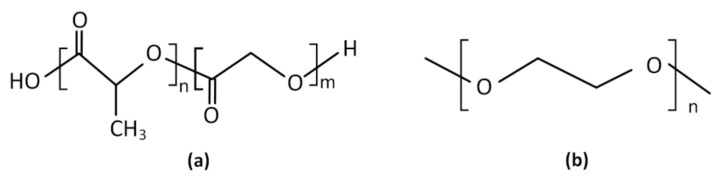
Structures of: (**a**) poly(lactic-co-glycolic acid) (PLGA); (**b**) poly(ethylene glycol) (PEG).

Finally, another promising drug delivery system utilizing advanced polymeric materials for the treatment of xerostomia was that of Muthumariappan et al. [138], who developed a localized formulation consisting of pilocarpine-loaded poly(lactic-co-glycolic acid) (PLGA)/poly(ethylene glycol) (PEG) nanofiber mats via electrospinning. The selection of these polymers, whose structures are presented at Figure 6, was made according to their favorable biodegradability and biocompatibility. Results showed that within the first 24 h of the application, pilocarpine-loaded nanofiber mats had a higher saliva secretion compared to the conventional systemic pilocarpine.

## 8. Summary and Conclusions

Oral dryness is a complex condition expressed as a physiological deficiency with or without perceived dysfunction. Xerostomia is most commonly presented in patients treated with certain medications, those subjected to head and neck radiotherapy, or in individuals with specific systemic diseases, such as Sjögren’s syndrome. Even though it mostly affects geriatric patients, xerostomia can also be observed in young individuals. The development of a successful treatment approach requires the establishment of the correct diagnosis, which encompasses the distinguishment of patients with subjective complaints from those presenting salivary gland hypofunction, and, subsequently, the identification of the underlying etiology. However, the complex nature and functions of saliva pose challenges that needs to be surpassed during the development of the management approach. Remedies for patients with hyposalivation and xerostomia are mainly directed at the relief of symptoms and the prevention of oral complications. In any case, based on the detailed literature survey conducted above, it is an indisputable conclusion that advanced polymeric materials play a vital role in the development of the various management approaches of xerostomia, concerning either already commercially available formulations or drug delivery systems currently under research and development.

## Figures and Tables

**Table 1:** Category of xerogenic medications and active pharmaceutical ingredients [42,45].

Category	Drug Substance
Antidepressant agents and antipsychotic agents	citalopram, fluoxetine, paroxetine, sertraline, venlafaxine, amitriptyline, imipramine, reboxetine, bupropion hydrochloride, clozapine, chlorpromazine, haloperidol, olanzapine
Anticholinergic agents	dicyclomine, mepenzolate
Antihypertensive agents	captopril, clonidine, methyldopa, prazosin
Antiparkinsonian agents	biperiden, selegiline
Diuretic agents	spirnolactone, chlorothiazide, furosemide, hydrochlorothiazide
Opioids	morphine, codeine, methadone, pethidine
Immunostimulants	interferon-alpha

**Table 2:** Commercially available saliva substitutes mentioned in published studies.

Dosage Forms	Brand Name	Polymers Used	Product Composition	Characteristics of the Formulation: Advantages or Disadvantages	Manufacturer	Ref.
Oral Sprays	Aldiamed^®^	CMC	Water, propylene glycol, xylitol, glycerol, microcrystalline cellulose, panthenol, CMC, sodium, sodium benzoate, lactoferrin, disodium EDTA, lysozyme, hydrochloride, aroma, Aloe Barbadensis	Significant improvement of xerostomia and increased life quality. Diminished use frequency, as compared to the other respective saliva substitutes, which may be associated to the improved results on mouth dryness.	Certmedica International	[93]
	Artisial^®^	Sodium CMC	Sodium CMC, sorbitol, calcium chloride dihydrate, magnesium chloride, dipotassium phosphate, monopotassium phosphate, potassium chloride, sodium chloride	Only minimal enamel mineral loss was observed in relevant published studies.	Jouveinal Laboratoires	[94]
	Aqwet^®^	CMC	Water, CMC, sorbitol, potassium chloride, sodium chloride, magnesium chloride, calcium chloride	Improved wetting ability as compared to similar commercially available saliva substitutes; comparable properties with human saliva.	Cipla Ltd. (Mumbai, India)	[95]
	Biotene^®^	Xanthan gum	Water, glycerin, xylitol, PEG-60, hydrogenated castor oil, VP/NA copolymer, sodium benzoate, Xanthan gum, methylparaben, propylparaben sodium saccharin, cetylpyridinium chloride, limonene	Effective in reducing mouth dryness, taste alteration, and chewing difficulties. Not well-tolerated and limited acceptance from patients.	GlaxoSmithKline	[96]
	EMOFLUOR^®^	HEC	Water, glycerin, sorbitol, maltitol, ammonium phosphate, HEC, ammonium fluoride, methylparaben, sodium saccharin, sodium chloride, potassium chloride, propylparaben	Erosion-protective potential, which may be connected to the product’s film-forming properties.	Dr. Wild&Co AG	[93]
	Entertainer^®^	CMC	Water, CMC, aloe vera, glycerin, dibasic sodium phosphate, potassium chloride	High popularity among performers and voice clinicians; has gained increased interest as possible laryngeal lubricants due to quick throat comfort and vocal quality improvement. However, it has a relatively short-term effect.	KLI Corporation (Carmel, IN, USA)	[97]
	Glandosane^®^	Sodium CMC	Potassium chloride, sodium chloride, magnesium chloride, Magnesii chloridum, calcium chloride, potassium monohydrogen phosphate, sodium CMC, sorbitol	Preferred by patients due to the good taste and the easy handling. However, it has revealed a high demineralizing potential in several in vitro studies.	Helvepharm	[98]
	Oasis^®^	Copovidone	Cetylpyridinium chloride, copovidone, flavor, methylparaben, PEG-60 hydrogenated castor oil, propylparaben, sodium benzoate, sodium saccharin, water, xanthan gum, xylitol	Significantly reduced enamel loss as compared to a positive control.	Oasis Consumer Healthcare	[99]
	Stoppers 4^®^	HEC	Water, glycerin, xylitol, HEC, lysozyme, lactoferrin, glucose oxidase, spearmint (natural), sodium benzoate	Increased enamel loss as compared to a positive control.	Jocott Brands Inc. (Van Nuys, CA, USA)	[93]
Oral Solutions	Act^®^	Poloxamer		Provides immediate but not long-lasting effect.	Sanofi	[40]
	Orazyme	Poloxamer and Sodium CMC	Gluconate, aloe Barbadensis, sodium CMC, poloxamer, water	Similarly with the abovementioned oral solution, it fails to provide long-lasting effect.	Dr. Fresh	[100]
	Xeros^®^	HEC	HEC, betaine, xylitol, sodium fluoride, water, allantoin	Decreases the patients’ discomfort during night but presents more significant effects in patients whose residual secretory potential was severely compromised.	Dentaid	[101]
Gels	Biotene oralbalance	HEC	Lactoperoxidase, lysozyme, glucose oxidase, lactoferrin, hydrogenated starch hydrolysate, xylitol, HEC, glyceryl polymethacrylate beta-D-glucose, aloe vera, potassium thiocyanate	Significant improvement in dryness, swallowing, and taste. Low retention time, which may be attributed to the relatively low viscosity.	GlaxoSmithKline	[87,102,103]
	OralSeven	HEC	Hydrogenated starch hydrosylate, glycerin, water, xylitol, glyceryl acrylate, acrylic acide copolymer, HEC, aloe barbadenisis, lactoperoxidase, dextrose monohydrate, glucose oxidase, lactoferrin, lysozyme, potassium thiocyanate, cellulose gum	Considerable problems with the application and the handling of the gel were referred by patients.	Oral7 International	[3]
Lozenges	Salese	Ethylcellulose and xanthan gum	Ethyl cellulose, xanthan gum, xylitol, sodium bicarbonate, eucalyptus oil, wintergreen oil, glycerol, zinc gluconate, thymol, calcium sulfate, potassium phosphate dibasic	Significantly low erosive potential on enamel, probably due to formulation’s high pH. However, the efficacy and patients’ acceptance of higher pH products are not yet known.	Nuvora Inc. (Santa Clara, CA, USA)	[56,104]
	SalivaSure^®^	CMC	Xylitol, malic acid, dibasic calcium phosphate, CMC, sodium citrate dihydrate, stearic acid, citric acid, magnesium stearate, silica colloidal	Xylitol contained in the formulation reduces plaque formation and minimizes dental caries. Furthermore, no interaction with prescription medications has been reported, and the formulation is regarded as safe for people with diabetes. Main drawback is the short-lasting relief on contact.	Scandinavian Formulas Inc. (Sellersville, PA, USA)	[102,103]

**Table 3:** Polymers used in chewing gums’ formulations.

Polymer	Examples	Ref.
Natural polymers	Polymers based on glycerol	[114]
Synthetic polymers	Polyisobutylene	[101,115,116,117]
	Isoprene copolymer	[118]
	Styrenebutadiene copolymers	[115,116,117]
	Polyvinyl acetate	[118]
	Polyvinyl alcohol	[114,119]

**Table 4:** Commercially available chewing gums used in xerostomia.

Product Name	Characteristics	Manufacturer	Ref.
Freedent White^TM^	As a low-tack chewing gum, it provides a better tolerance in patients with dental prostheses as compared to the normal-tack chewing gums. Nevertheless, several adverse effects (i.e., irritation of mouth, nausea etc.) have been reported.	Wrigley company	[108,115]
V6 chewing gum	Acceptable consistency and no reports of mouth irritation.	Gadbury	[116,120]
Dentirol chewing gum	Satisfying taste and acceptable consistency. Alleviates the symptoms without increasing the saliva flow rate.	Continental Candy Company, Denmark	[117]
Xerostom Chewable Relief Capsules^®^	Improves speech, swallowing; decreases subjective xerostomia.	Biocosmetics laboratories, Spain	[121]
Biotene chewing gum	Xylitol contained in the formulation reduces plaque formation and minimizes dental caries; improved results when combined with the respective oral solution and mouth paste.	GlaxoSmithKline	[122]

## Data Availability

Not applicable.

## Abbreviations

BMI	Body mass index
CMC	Carboxymethyl cellulose
DMFT	Decayed missing filled teeth
HEC	Hydroxyethyl cellulose
HM-EHEC	Hydrophobically modified ethyl hydroxyethyl cellulose
HPMC	Hydroxypropylmethyl cellulose
LM-pectin	Low-methoxylated pectin
MC	Methyl cellulose
MNA	Mercaptonicotinic acid
OHRQOL	Oral health-related quality of life
PEG	Poly(ethylene glycol)
PEO	Poly(ethylene) oxide
PLGA	Poly(lactic-co-glycolic acid)
PPG	Poly(propylene glycol)
SCMC	Sodium carboxymethyl cellulose
SWS	Stimulated whole saliva
UWS	Unstimulated whole saliva
